# Optimization of Biocomposite Film Based on Whey Protein Isolate and Nanocrystalline Cellulose from Pineapple Crown Leaf Using Response Surface Methodology

**DOI:** 10.3390/polym14153006

**Published:** 2022-07-25

**Authors:** Fitriani Fitriani, Sri Aprilia, Muhammad Roil Bilad, Nasrul Arahman, Anwar Usman, Nurul Huda, Rovina Kobun

**Affiliations:** 1 Doctoral Program, School of Engineering, Post Graduate Program, Universitas Syiah Kuala, Banda Aceh 23111, Indonesia; fitriani18@mhs.unsyiah.ac.id; 2 Department of Chemical Engineering, Universitas Syiah Kuala, Banda Aceh 23111, Indonesia; nasrular@unsyiah.ac.id; 3 Faculty of Integrated Technologies, Universiti Brunei Darussalam, Bandar Seri Begawan BE1410, Brunei; roil.bilad@ubd.edu.bn; 4 Department of Chemistry, Faculty of Science, Universiti Brunei Darussalam, Jalan Tungku Link, Bandar Seri Begawan BE1410, Brunei; anwar.usman@ubd.edu.bn; 5 Faculty of Food Science and Nutrition, Universiti Malaysia Sabah, Kota Kinabalu 88400, Sabah, Malaysia; rovinaruby@ums.edu.my

**Keywords:** biocomposite film, whey protein isolate, nanocrystalline cellulose, response surface methodology, physical properties, mechanical properties

## Abstract

This study employed response surface methodology to optimize the preparation of biocomposites based on whey protein isolate, glycerol, and nanocrystalline cellulose from pineapple crown leaf. The effects of different concentrations of nanocrystalline cellulose as a filler and glycerol as a plasticizer on the thickness, the tensile strength, and the elongation at break on the resulting biocomposite films were investigated. The central composite design was used to determine the optimum preparation conditions for biocomposite films with optimum properties. The regression of a second-order polynomial model resulted in an optimum composition consisting of 4% glycerol and 3.5% nanocrystalline cellulose concentrations, which showed a desirability of 92.7%. The prediction of the regression model was validated by characterizing the biocomposite film prepared based on the optimum composition, at which the thickness, tensile strength, and elongation at break of the biocomposite film were 0.13 mm, 7.16 MPa, and 39.10%, respectively. This optimum composition can be obtained in range concentrations of glycerol (4–8%) and nanocrystalline cellulose (3–7%). Scanning electron microscope images showed that nanocrystalline cellulose dispersed well in the pure whey protein isolate, and the films had a relatively smooth surface. In comparison, a rough and uneven surface results in more porous biocomposite films. Fourier transform infrared spectroscopy revealed that nanocrystalline cellulose and glycerol showed good compatibility with WPI film by forming hydrogen bonds. The addition of nanocrystalline cellulose as a filler also decreased the transparency, solubility, and water vapor permeability and increased the crystallinity index of the resulting biocomposite film.

## 1. Introduction

The development of biodegradable materials for food packaging has been significantly increasing due to the various environmental problems associated with synthetic materials derived from petroleum [1]. Biopolymer materials extracted from biomass are generally classified into three groups, polysaccharide, protein, and lipid, and are the biodegradable materials in focus [2], due mainly to their status as low-cost, renewable, abundant, and easily accessible [3]. These materials can also be extracted from agricultural byproducts or waste, increasing their added value and providing significant economic and environmental benefits [4]. Particularly, whey protein isolate (WPI), a valuable by-product of cheese and casein production, consisting of more than 90 wt% protein, is an attractive biopolymer for food packaging applications because of several benefits, such as biodegradability, good film-forming properties, biocompatibility, and abundance in nature [5,6]. Previous studies have been conducted using other proteins as biodegradable materials to develop the packaging film that can be obtained from plant, animal and microbial sources [4,7,8,9]. WPI-based films also provide functional properties, such as transparency, flexibility, and an excellent barrier against oxygen permeation, which are attractive for food packaging applications [10,11,12]. However, the hydrophilic nature of WPI-based films and their low mechanical and high water vapor permeability as biodegradable materials limited their ability to reach broad commercial applications [13,14,15].

Several strategies have been used to improve the properties of WPI-based films, including the incorporation of biomaterials, nanomaterials, plasticizers, and other crosslinking agents to form biocomposite films that incorporate the advantages of biopolymers while minimizing their drawbacks [16,17]. Numerous studies on WPI (protein) based film with the incorporation of nanomaterials such as nanocrystalline cellulose have demonstrated vast improvements on the mechanical properties of the resulting biocomposite films [18,19,20].

Due to its sustainability, availability, lightness, large surface area, and high mechanical strength, nanocrystalline cellulose (NCC) has attracted a great deal of interest as a potential nano-reinforcement for WPI-based films [18,21]. NCC is a nanometer-sized crystal particle produced as a stable aqueous colloidal suspension and is typically isolated from cellulose sources through acid hydrolysis [22]. Isolation of NCC was reported from different sources such as sugarcane baggases [21], rice straw [23], kenaf bast [24], conorcapus fiber [25], walnut shell [26], and pineapple crown leaf [22].

Pineapple is a common fruit which is widely used as a source of food production. The global market for pineapple and its products has shown a significant increase [22]. Annually, pineapple processing generates 3 billion tons of by-products such as pineapple crown leaf, which causes environmental issues [27]. In several cases, pineapple crown leaves are burned or disposed of in landfills with no regard for the environment. Hence, suitable methods for converting pineapple crown leaves to upgraded products must be implemented.

Pineapple crown leaf fibers were identified as an excellent source of cellulose [28]. Pineapple crown leaves contain 79–83% cellulose, 29% hemicellulose, and 5–15% lignin [29]. In our previous study, the use of pineapple crown leaves as the source of nanocrystalline cellulose (NCC) offered interesting properties that can be used in several applications, which was proven by an improvement to the physical and mechanical properties of whey protein film [18]. Due to this advantage, NCC derived from pineapple crown leaf can partially replace the conventional synthesized polymer packaging materials, while achieving environmental standards. Furthermore, adding plasticizers such as glycerol to the biocomposite film can improve the toughness and minimize brittleness while enhancing polymer chain mobility [30]. Zhai et al. recently reported that adding glycerol could enhance the ductility and elasticity of a WPI film [31].

When preparing a biocomposite film, it is critical to determine the optimum concentrations of the casting solution film compositions to achieve the required composite film properties. Central composite design (CCD) using response surface methodology (RSM) is commonly used to attain this objective. RSM is a mathematical and statistical technique that provides optimal conditions by analyzing the relative importance of all influencing factors [32]. Furthermore, the mathematical models generated by RSM can reveal the interactions between the components of a biocomposite film and its properties by fitting mathematical models from the analysis of variance (ANOVA) [33]. Recent studies have used RSM to achieve optimal formulation in biocomposite film properties such as soy protein films with the addition of glycerol [34], corn starch–chitosan films with the addition of starch nanocrystals [35], and zein films with the addition of gelatin and glutaraldehyde [36]. In this study, the optimization of WPI-based film with the addition of glycerol and nanocrystalline cellulose from pineapple crown leaf using RSM was investigated. The objective of this study was to determine the optimization or the best design parameters of WPI-based film with incorporations of different range concentrations of glycerol (4–8%) and NCC (3–7%) from pineapple crown leaf using RSM-CCD and to investigate the physical and mechanical properties of prepared films.

## 2. Materials and Methods

### 2.1. Materials

Whey protein isolate containing 90% protein was purchased from Glanbia Nutritionals (Carlsbad, California, United States). Glycerol, sodium hydroxide (NaOH), hydrogen peroxide (H_2_O_2_), and sulfuric acid (H_2_SO_4_) were obtained from Merck (Darmstadt, Germany). The NCC was prepared from the isolation of pineapple crown leaf waste using the acid hydrolysis method according to the protocol established earlier [22].

### 2.2. Isolation of Nanocrystalline Cellulose

**Figure 1:**
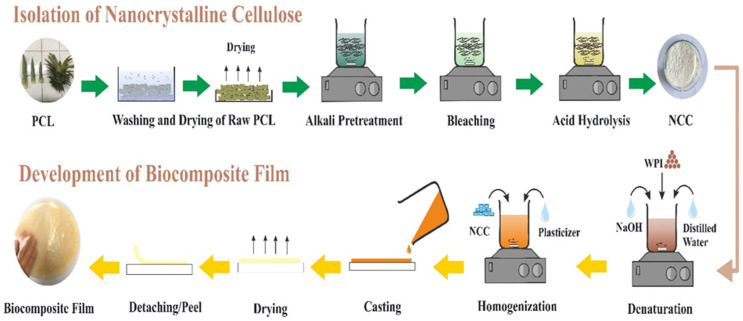
Schematic illustration of nanocrystalline cellulose isolation and biocomposite film preparation.

The pineapple crown leaf (PCL) waste was first washed and dried as illustrated in Figure 1. Pre-chemical treatment and hydrolysis were used to isolate the NCC according to a method reported earlier [22]. Firstly, the dried PCL was ground and sieved under 40 mesh. The ground fibers were treated in 1 M NaOH and 1 M H_2_O_2_ at 80 °C for 1 h to perform the alkali and bleaching treatment. The fiber residue from the alkali and bleaching treatments was washed several times with distillate water until it reached a neutral pH. Acid hydrolysis was then carried out using 1 M H_2_SO_4_ for 3 h at 45 °C to produce NCC particles through the depolymerization process. The distilled water was added to resulting solution and cooled in a water bath at 20 °C for 24 h to stop the acid hydrolysis. The suspension was neutralized by centrifugation at 2000 rpm for 30 min with repeated additions of distilled water, followed by ultrasonication for 30 min to remove the excess acid. The NCC product was then dried and ground into powder before being stored at room temperature until further use.

### 2.3. Preparation of Whey Protein/Nanocrystalline Cellulose/Glycerol Biocomposite Films

Biocomposite films were produced via the solution casting method reported earlier by Fitriani et al. [14] with a few minor modifications, as illustrated in Figure 1. To hydrate the proteins, 6% (*w*/*v*) WPI solution was prepared in 200 mL distilled water at pH 8 (pH was regulated with NaOH) using a mechanical stirrer for 30 min at room temperature. The WPI solution was then immersed in a 90 °C water bath with constant stirring for 30 min to denaturize the protein. The WPI solution was then mixed with glycerol of varying amounts (4%, 6%, and 8% *w*/*w*) and NCC of varying amounts (3%, 5%, and 7% *w*/*w*) using a mechanical stirrer at room temperature for 30 min with a speed of 250 rpm. The WPI and WPI/NCC solution films were poured into 20 cm × 15 cm silicon mold and dried by oven drying at 60 °C for 24 h. All biocomposite films were stored at room temperature until analysis.

### 2.4. Physical and Mechanical Properties

For each film sample, the thickness was measured using a micrometer with a 0.001 mm accuracy at five different random areas. Using the average thickness value, the mechanical parameters of the film sample were calculated. The mechanical characteristics of the biocomposite films were determined using an MTS Exceed Universal Testing Machine E43 (Eden Prairie, MN, USA) according to the ASTM standard method D638 Type IV.

### 2.5. Morphological, Physical, and Crystallinilty Analysis of Optimized Biocomposite Films

A scanning electron microscope was used to examine the surface and cross-sectional morphology of the biocomposite films (SEM, JEOL JSM-6360OLA, Tokyo, Japan). The films were coated with a thin layer of gold before being observed at 14 kV. A Fourier transform infrared spectrophotometer was used to measure the functional groups of the films (FTIR, IRPrestige-21, Shimadzu, Kyoto, Japan). The spectra of the films were obtained using 25 scans at a resolution of 4 cm1 for each sample, with scanning ranges ranging from 4000 to 400 cm^−1^. The transparency of the optimized films was analyzed using a UV-Vis spectrophotometer (UV-1700, Shimadzu, Kyoto, Japan) at a wavelength of 300–800 nm according to ASTM D1746-09 standard. The water solubility (WS) of films was measured using the following procedure. Films with dimensions of 3 cm × 3 cm were dried in an oven at 50 °C for 5 h followed by weighing to measure the initial dry weight. The films were then immersed in 40 mL of distilled water for 24 h at room temperature. The final dry weight was obtained after the insoluble films were dried in an oven at 110 °C for 5 h, and the water solubility (WS, %) of the films was then determined according to Equation (1).

(1) $$\mathrm{WS}\;\left(\%\right)=\frac{\mathrm{Initial}\;\mathrm{dry}\;\mathrm{weight}\;-\;\mathrm{Final}\;\mathrm{dry}\;\mathrm{weight}}{\mathrm{Initial}\;\mathrm{dry}\;\mathrm{weight}}\;\times 100$$

The water vapor permeability (WVP) of the films was determined using the gravimetric method, followed by a modified procedure of the ASTM E96 standard [37]. The films were sealed in an 8 mm diameter glass permeation cup. Then, the cups were filled with distilled water and placed in a desiccator to achieve 100% air humidity. Over 48 h, the cup was weighed every 3 h. The water vapor permeability (WVP, g/m.s.Pa) was calculated according to Equation (2).

(2) $$\mathrm{WVP}=\frac{C\times T}{A\times \Delta P}$$

where C is the water vapor transmission rate (g/s), T is the thickness of the films (mm), A is the area of exposed film (mm^2^), and ΔP is the difference in water vapor pressure all over the films (Pa). The crystallinity of the film was analyzed using an X-ray diffraction Shimadzu XDR 7000 (Japan) with CuKα radiation (λ = 0.1541) operating at 40 kV and 30 mA. The 2θ angle was scanned in the range of 10–70°. The crystallinity index (CI, %) was calculated using the Segal method, as given by Equation (3) [38].

(3) $$\mathrm{CI}\;\left(\%\right)=\frac{I_{200}-{\;I}_{\mathrm{am}}}{I_{200}}\;\times 100$$

where I_200_ is the maximum intensity of the largest crystal plane reflection (2θ = 22°) and I_am_ is the minimum intensity of the diffraction at 2θ = 9.5°.

### 2.6. Experimental Design and Statistical Analysis

The formulation for the fabrication and optimization of biocomposite films was designed using RSM based on CCD. Design Expert 11 (Stat-Ease Inc., Minneapolis, MN, USA) was used to create the experiment design. The two independent variables were the glycerol concentrations (X_1_) and NCC concentrations (X_2_). Four axial experiments of levels ±α, eight factorial experiments of levels ±1, and four replicates in the center point were included in the design. The level of factors and the code of variables are shown in Table 1.

The design matrix for biocomposite films is shown in Table 2. The response functions for thickness, tensile strength, and elongation at break were evaluated. For each factor of responses, a second-order quadratic model as a function of X was fitted using Equation (4):

(4) $${Y=\beta }_{0}+\sum_{i=1}^{3}\beta_{i}X_{i}+\sum_{i=1}^{3}\beta_{\mathrm{ii}}X_{1}^{2}+\sum_{i=1}^{3}\beta_{\mathrm{ij}}X_{i}X_{j}$$

where Y is the predicted response, β_0_ is the constant, β_i_ represents the coefficients variable for linear terms, β_ii_ denotes the coefficients variable for quadratic terms, β_ij_ represents the coefficients variable for interactive terms, and X_1_ and X_2_ are the independent variables of glycerol and NCC concentrations, respectively. The variation of each factor was classified into three categories, namely linear, quadratic, and interactive. The relevance of these variables and the adequacy of the second-order quadratic function were determined using the components’ lack-of-fit and error.

## 3. Results and Discussion

### 3.1. Thickness, Tensile Strength, and Elongation of Biocomposite Films

Table 3 summarizes the results of the 13 conducted experiments used to determine the thickness, tensile strength, and elongation of the biocomposite films prepared based on the optimum composition suggested by RSM. The concentrations of glycerol and NCC were the two tested factors. The thickness of the biocomposite film was found to be in the range from 0.07 to 0.24 mm. The tensile strengths of biocomposite films were 1.64–7.85 MPa., and the elongation at break was 4.71–42.92%. The highest thickness (0.24 mm) was obtained for the biocomposite film containing 8% glycerol and 7% NCC. The highest tensile strength was 7.85 MPa at 3.17% glycerol and 5% NCC. Meanwhile, the highest elongation was at 42.92% at 6% glycerol and 2.17% NCC.

In general, the crystal formation can occur when natural fibers are utilized as the filler materials in biopolymer matrices like NCC, limiting the physical and mechanical properties of the biocomposite films [21,39]. The thickness, tensile strength, and elongation at break of the prepared biocomposite films were analyzed using RSM-CCD, and the impacts of the independent factors on the responses are further discussed below.

### 3.2. Model Selection and Verification of Thickness, Tensile Strength, and Elongation

To test the significance of the coefficient terms, all the response variables of biocomposite films were analyzed using ANOVA and regression analysis for model fitting. Table 4, Table 5 and Table 6 show the ANOVA for the quadratic model responses for the thickness, the tensile strength, and the elongation at break parameters, respectively.

The ANOVA test revealed that the quadratic regression models of the thickness, the tensile strength, and the elongation at break were highly significant (*p* < 0.05). This probability value indicates that the F-value was extremely unlikely to occur because of the noise [40]. Meanwhile, the lack of fit test provides information on the adequacy of the fitted model by measuring the error caused by a defect in the model [41]. The findings revealed that the *p*-values for lack of fit were non-significant (*p* > 0.05) for all responses, indicating that the model was well-fitting and sufficient for data explanation. The opposite of lack-of-fit of the model for biocomposite films was further investigated using the R^2^ value. The results showed that *R*^2^ of the biocomposite films model for the thickness, the tensile strength, and the elongation at break were 0.9054, 0.9408, and 0.9455, respectively. Additionally, the adequate precision value was significantly greater than 4 for all responses, indicating that all response surface models had acceptable values. Based on those statistical analyses, the developed experimental models are suitable for predicting the physical and mechanical properties of biocomposite films within the experimental limits in this study.

The *p*-values for each response from biocomposite films are presented in Table 7. It was indicated that the model terms had a significant effect on the variable responses in biocomposite film properties. The glycerol (X_1_), NCC (X_2_), interaction of glycerol and NCC (X_1_X_2_), two-level interaction of glycerol (X_1_^2^), and two-level interaction of NCC (X_2_^2^) were all significant (*p* < 0.05) for the thickness and tensile strength properties. However, the interaction of glycerol and NCC (X_1_X_2_) was not significant for the elongation property, whereas the other model terms were found to be significant. This can occur because a higher regression value directly interprets a more significant impact of the independent variables in biocomposite films on the variable responses of film properties [42].

For all responses, glycerol and NCC gave the highest regression coefficient values. As a result, glycerol and NCC had the most significant impact on the physical and mechanical properties of the biocomposite films. Furthermore, the positive coefficients for the glycerol and NCC variables indicated a significant effect on the properties of the film. The negative coefficients for the two variables, on the other hand, show an excellent dividing effect on the properties of the biocomposite films [41]. The biocomposite films fit the quadratic regression model better for thickness, tensile strength, and elongation at break. Equations (5)–(7) in terms of the coded values represent the estimated models built for the thickness, tensile strength, and elongation at break methods, respectively. However, it should be acknowledged that the following equations are only limited within the range of tested glycerol concentration (4–8%) and NCC concentration (3–7%).

(5) $$Y_{1}=0.2064+0{.0235X}_{1}+0{.0328X}_{2}+0.0364\mathrm{XY}-{0.0430X}_{1}^{2}-{0.0352X}_{2}^{2}$$

(6) $$Y_{2}=4.38-{1.83X}_{1}-{0.4423X}_{2}+0.9812\mathrm{XY}+{0.2399X}_{1}^{2}-{0.9858X}_{2}^{2}\;$$

(7) $$Y_{3}=35.41-{10.59X}_{1}-{9.21X}_{2}+2.51\mathrm{XY}-{7.47X}_{1}^{2}-{4.50X}_{2}^{2}$$

where Y_1_, Y_2_, and Y_3_ are the predicted response for thickness, tensile strength, and elongation at break, X_1_ is glycerol concentration, and X_2_ is NCC concentration. The regression between the model and experimental data were for thickness, tensile strength, and elongation at break indicate that the quadratic model fits are acceptable, with *R*^2^ values being 0.9054, 0.9408, and 0.9455, respectively. This value indicated that the models could not explain only 9.46% of the thickness, 5.92% of the tensile strength, and 5.45% elongation at break variation.

### 3.3. Analysis of Response Surface of Thickness, Tensile Strength, and Elongation

**Figure 2:**
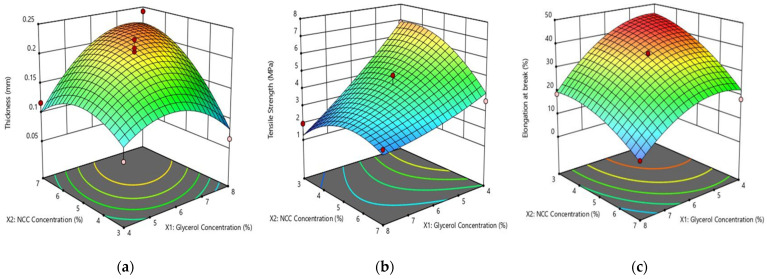
Response surface plot of (**a**) thickness; (**b**) tensile strength; and elongation (**c**) as influenced by the addition of glycerol and NCC.

Figure 2 shows the 3D response surface and contour plots of the effect of independent variables of glycerol and NCC concentrations on thickness, tensile strength, and elongation at break using Equations (3)–(5). The 3D response surface was obtained by varying the two variables of glycerol and NCC. It can be clearly seen that the concentrations of glycerol and NCC have a quadratic effect on the physical and mechanical properties of the biocomposite films. Figure 2a demonstrates that the concentration of glycerol and NCC has a significant effect on thickness. When glycerol concentration rises from 4% to 6% and NCC concentration rises from 3% to 5%, the thickness increased in a parabolic trend. Furthermore, increasing the NCC and glycerol concentrations resulted in thicker biocomposite films. The maximum thickness of 0.24 mm was observed at 8% glycerol and 7% NCC and minimum thickness of 0.07 mm was observed with 3.17% glycerol and 5% NCC. This increase in film thickness can be attributed to the distribution of NCC as a filler and a higher content of glycerol in the resulting film [18]. These results are similar to a previous study of the effect of addition glycerol and NCC on the soy protein isolate and WPI [18,34].

Figure 2b shows the effects of varying glycerol and NCC concentrations on the tensile strength of biocomposite film. It was found that an increase in glycerol concentrations decreases the tensile strength. This can be rationalized by considering that glycerol with low molecular weight generates molecular interactions along polymer chains, increasing film flexibility while decreasing tensile properties. It would also reduce intramolecular hydrogen bonding in the network of the film. This result was similar to the chitosan–NCC film and gelatin film with the addition of glycerol [43,44]. Furthermore, glycerol acted as a plasticizer in the composite film matrix, resulting in an increased free volume and reduced network rigidity in WPI polymers. Plasticization also led to the composite films’ flexibility, reducing the tensile strength [45]. The NCC content also affected the tensile strength of the composite film, as higher NCC increased the tensile strength of the film. This result was consistent with our previous study, which used NCC as a filler in a WPI-based film [18]. The reinforcement process to increase the tensile strength by incorporating rigid nanoparticles into a polymer matrix was also ascribed to the strong interfacial interaction between NCC and WPI matrix, which resulted in the effective transfers of stress from the polymer matrix to the nanoparticles that effectively carry the load and enhance the strength of the composite films [46,47].

Figure 2c shows that higher glycerol and NCC concentrations decreased the elongation at break of the biocomposite film. The trend could be attributed to glycerol and NCC hydrophilicity, as well as the degree of thermal degradation of the NCC. The high shear stress of the polymer decreased its flexibility of films during the blending of the composite with the natural fillers, which causes the limitation of molecular chain motion in the polymer. NCC also caused non-homogeneous film matrices, which gave more dispersed granules in the films which in turn disrupted the interaction between the networks and strongly affected the elongation [48]. As the filler and plasticizer content increased, there was a probability of this phenomenon occurring during blending which would enhance the interaction between the filler and the polymer surface resulting in shorter fibers than critical grades and a reduced aspect ratio. This then directly affected the resulting composite performance and decreased tensile strength and elongation [45]. These results are also in agreement with previous studies of chitosan, sesame protein, and faba bean protein film [43,49,50].

### 3.4. Optimization Process of the Experiment

Glycerol and NCC concentration are the two factors in selecting optimization to make the desired composition in this study. After optimization of the experiment, there are several solutions for the physical and mechanical properties the biocomposite films, as shown in Table 7. As a result, the response was the highest obtained from the experiments. A value close to one (100%) is an acceptable value for the desirability function. The desirability function sets perimeters to find the optimal value for all the variable responses during the optimization procedure. In this study, the optimum physical and mechanical properties of the biocomposite with a desirability of 92.7% were associated with 4% glycerol and 3.5% NCC concentration. The level of this independent variable resulted in the highest thickness, tensile strength, and elongation responses, which were 0.12 mm, 6.97 MPa, and 44.65%, respectively. This study showed that the addition of high glycerol and NCC decreased the thickness, tensile strength, and elongation of the biocomposite film. Aprilia et al. also reported that the irregular structure of the reinforcing material reduced composite strength because of the bonds’ inability to support the stress transfer of the polymer matrix [51]. The weakness in the interfacial areas would decrease the effect of stress transfer from the polymer matrix to the filler component, lowering the strength of the films [39]. These results also show similarities with the study conducted by Kusmono *et al.*, where they discovered that the strength of the composite decreased as the amount of support material increased [43].

### 3.5. Validation Process of the Predicted Model of Optimised Formulations

To validate the model, an experiment was carried out with three replicates for each response under the optimal conditions suggested (4% glycerol and 3.5% NCC concentration). Table 8 shows the validation of the predicted model results, which show that the thickness, tensile strength, and elongation of biocomposite films were all within the predicted range at 0.13 mm, 7.16 MPa, and 39.10%, respectively. The 95% confidence interval was found to be within the validation results for all responses. A confidence interval estimate is a set of values that the parameter is almost definitely predicted to fall inside. The confidence interval indicates that the average data for all responses will decline within this range 95% of the time. In addition, the outcomes of all responses (thickness, tensile strength, and elongation) were within the 95% prediction interval value. Due to the additional uncertainty and scattered data involved in predicting a single response as well as the mean response, the prediction interval was greater than the confidence interval. It follows that the results could be 95% certain that they included all of the responses related to these settings. Overall, the model’s validity demonstrated that preparation of WPI-based composite films under the optimum suggested glycerol and NCC compositions by RSM was achieved.

### 3.6. Morphological, Physical and Crystallinilty Analysis of Optimized Biocomposite Films

#### 3.6.1. Functional Group of Optimized Biocomposite Film

**Figure 3:**
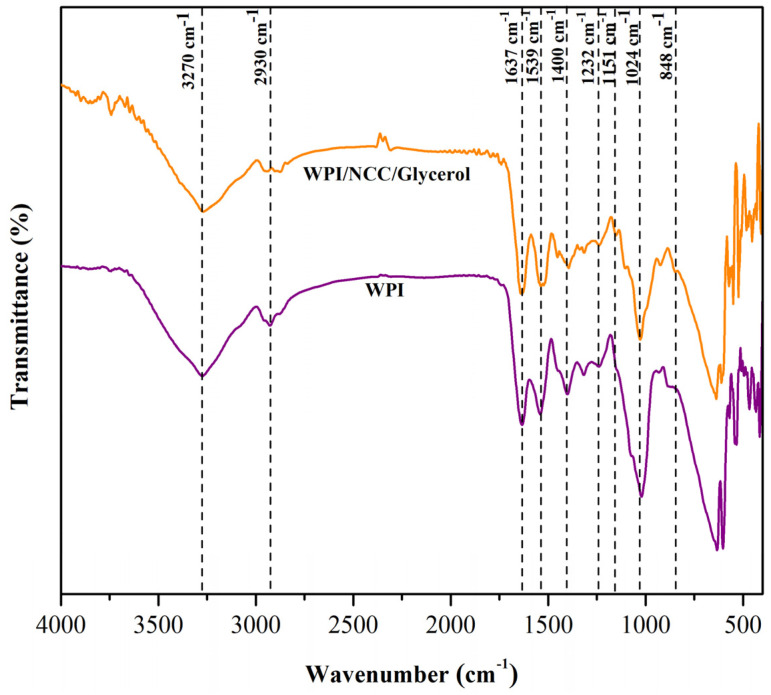
FTIR spectra of pure whey protein film and biocomposite film.

The FTIR spectra of the biocomposite films were obtained in order to estimate the functional groups of the films and analyze the interactions between the film components. Figure 3 shows the biocomposite film absorptions that occurred on bands ranging from 4000 to 400 cm^−1^. The biocomposite films exhibited a band at approximately 3270 cm^−1^, which is related to the presence of the N-H bond from WPI, the stretch of the OH groups, the presence of water in all films, and the presence of OH groups on the NCC surface [12,52]. The absorption band around 3270 cm^−1^ shifted to a lower wavenumber as the NCC remaining in the protein sample increased. With the addition of NCC content, the peaks displayed increased in intensity. These findings indicated that the NCC addition resulted in inter- or intramolecular hydrogen bonds as well as molecular rearrangement [12]. The band at 2930 cm^−1^ corresponds to the C-H group’s stretching vibration and is associated with a decrease in peak intensity, incorporating primary and secondary carbon bonds (CH_3_ and CH_2_). The biocomposite films retain the main absorption of WPI films, such as C=O stretching at 1637 cm^−1^ (amide I), N-H bending at 1539 cm^−1^ (amide II), C-H deformation at 1400 cm^−1^, and C-N stretching (amide III) at 1232 cm^−1^ [10]. When compared to pure WPI film, the amide I and II peaks in biocomposite films showed a slight shift to lower wavenumber. It is implied that the polarity decreased as more hydrogen bonds formed between glycerol, NCC, and protein molecules.

Furthermore, the addition of NCC increased the intensity of the 1151–1024 cm^−1^ region (C-O stretching and O-H deforming vibration). Early literature found the addition of NCC to WPI film containing more O-H leads to enhanced absorbance in the region [53]. The peak at 1024 cm^−1^ also exhibits a small displacement because of the potential extra interactions between the plasticizer and the film structure. The peak from 1151 to 848 cm^−1^ attributed to absorption bands of glycerol produced peaks in the films, corresponding to the vibration of C-C and C-O bonds [54]. Comparing the spectra of pure WPI film and biocomposite films, the peak of amide I, II, and III bands were more shifted. This implies that the addition of glycerol affected the interaction between NCC and WPI in the film matrix. Generally, NCC showed good compatibility with WPI film by forming hydrogen bonds with polypeptide chains [55].

#### 3.6.2. Morphology of Optimized Biocomposite Film

**Figure 4:**
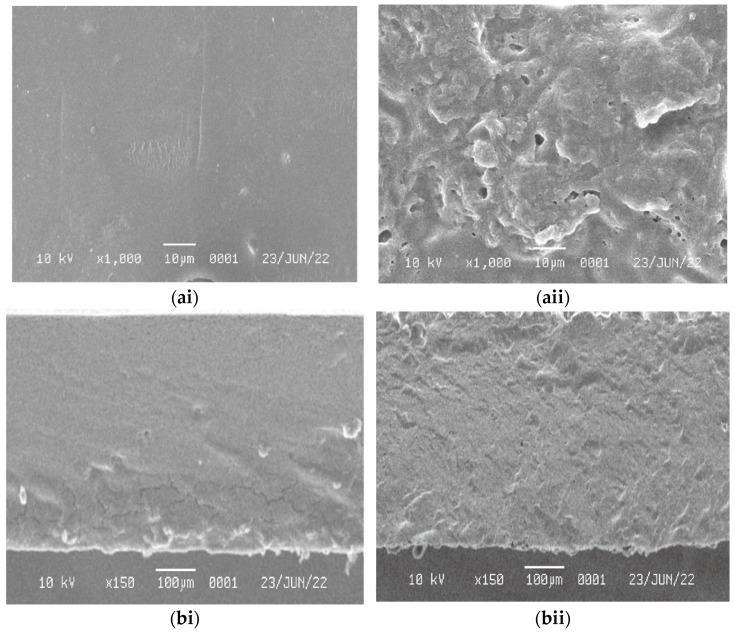
SEM image of (**a**) pure WPI film, and (**b**) biocomposite film on the surface (**i**) and cross section (**ii**).

The morphology of the WPI film and biocomposite films are shown in Figure 4. As observed, pure WPI film (Figure 4ai,aii) exhibited a smooth and homogenous structure. In contrast, the films containing NCC and glycerol (Figure 4bi,bii) had rougher surfaces with the presence of pores or cavities, which could be related to the formation of channels. It is clear that the addition of NCC as a filler causes significant changes in the film structure and increases the density of crack deflection sites in biocomposite films [56]. Due to the trapped air in the film solution, some pores or bubbles were probably created during the drying process of biocomposite films. NCC as filler and glycerol as plasticizer also acted as nucleating sites for voids growing. The protein denaturation process could also cause minor aggregation on pure WPI films. This experimental evidence is consistent with the findings of the previous study [57,58].

#### 3.6.3. Transparency of Optimized Biocomposite Film

Transparency of packaging materials portrays the products appearance and reflects the effect of NCC incorporation as a filler on the interaction matrix [53]. Table 9 shows the transparency of pure WPI film and biocomposite films. The biocomposite films were much less transparent than the pure WPI films across all wavelength ranges observed. This reduction indicates that the addition of NCC decreased the transparency of WPI films. At 800 nm, the transparency of pure WPI films was 22.5% and decreased to 13.4% with the addition of fillers. This could be due to the light path being blocked through the polymer matrix as a result of the strong interface interaction between the NCC and the WPI matrix [59]. Additionally, this decrease could be caused by the presence of the NCC particles, which increased the light scattering in the polymer matrix [10]. According to Qazanfadeh et al. [13] an NCC particle aggregation can affect the light transmission of biocomposite films by blocking the light’s passage through the protein film structure, confirming the SEM analysis results. Previous studies using a variety of biopolymer matrices, such as chitosan [43], alginate [60], casein [61], and starch [62], have observed a decrease in film transparency with the addition of cellulose nanoparticles. Furthermore, the presence of glycerol in WPI films might reduce the transparency of the biocomposite films as a result of the presence of a suitable contact area between the WPI, NCC, and glycerol structure [59].

#### 3.6.4. Water Solubility of Optimized Biocomposite Film

The water solubilities in biocomposite films are summarized in Table 10. This shows that the pure WPI films had a higher water solubility than the biocomposite films. The addition of NCC reduced the water solubility in WPI films. The decrease in solubility might be attributed to the formation of strong structures and bonds by the mixtures added to the protein matrix [18,63]. The NCC dimension and the crystalline area have been observed as effective characteristics in the water resistance of biocomposite films [64]. Water in the WPI film barrier also decreased due to the interaction between water and the hydroxyl groups of cellulose in NCC [10]. As previously reported, the use of NCC as filler at a lower concentration can reduce the water solubility in WPI films. This can be attributed to fewer nanoparticles being dispersed in the WPI matrix and the formation of NCC agglomerates [13]. Moreover, the hydrophilic nature of protein and glycerol as materials are the reasons for the higher solubility of pure WPI films in water than the biocomposite films with the addition of NCC [65].

#### 3.6.5. Water Vapor Permeability of Optimized Biocomposite Film

In the case of the developed biocomposite films, water vapor permeability is essential for innovative materials used for food packaging. The permeability results in Table 10 show that the addition of NCC and glycerol improved the WPI films barrier. Due to their hydrophilic nature, the water vapor barrier of protein polymer films is seen as a disadvantage of these materials. According to the previous studies, the incorporation of plasticizers and fillers in various types of micro and nanomaterials improves the biopolymers structure and characteristics [66]. When compared to pure WPI films, the addition of NCC reduced the water vapor permeability of the biocomposite films. The decrease in the water vapor permeability of the film could be attributed to NCC dispersion in the WPI polymer matrix, which favors the interference of water vapor diffusion [67]. Another possible explanation could be the hydrophilic nature of NCC as a filler in biocomposite films, reducing the water molecules adhesion [68].

The results obtained in biocomposite films appear to support those obtained in SEM analysis. This can be explained by the NCC interfering with the film morphology resulting in greater attraction and aggregation between the particles and causing the formation of less cohesive films, which may have contributed to water vapor permeation through the WPI matrix [69]. This improvement in WVP properties has previously been reported in studies using cellulose nanoparticles as a filler in whey protein-based film [10,13,37].

#### 3.6.6. Crystallinity of Optimized Biocomposite Film

**Figure 5:**
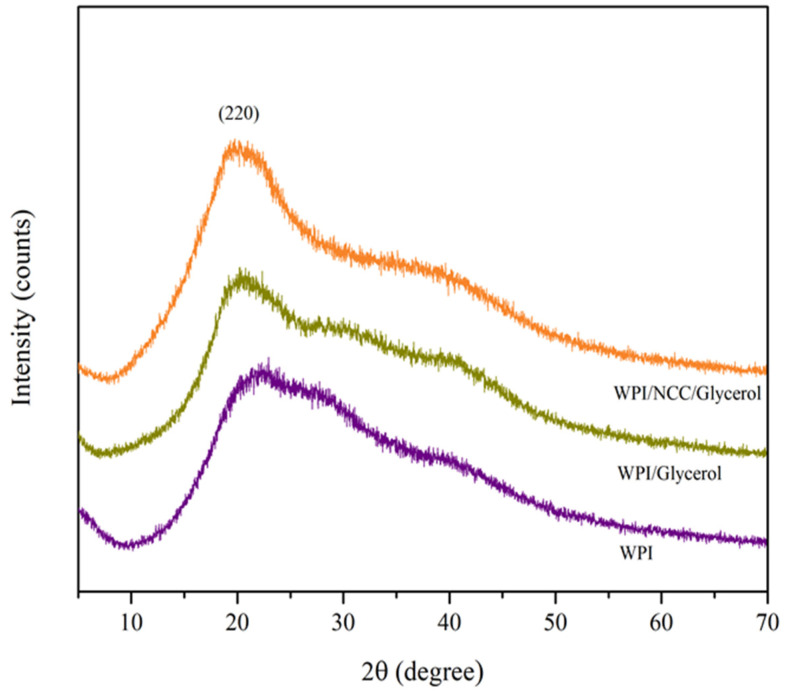
X-ray diffraction patterns of biocomposite film.

The X-ray diffraction patterns of films are related to the amorphous-crystalline structure and dependent on the crystallization rate in composite film. The XRD patterns of the pure WPI and optimal film samples are presented in Figure 5. A strong peak at around 2θ = 22° was observed in the diffractogram of the biocomposite films corresponding to the crystalline phase of (220) of the crystalline structure. The addition of glycerol and NCC resulted in similar peak locations, indicating that the morphology of the WPI crystalline structure remained unchanged [70]. Pure WPI also exhibited a broad peak at 20° and small peak at 30° to 40°, indicating the amorphous phase of the protein and also giving a relatively good crystalline degree, and can be classified as a semi-crystalline biopolymer. The semi-crystalline characteristic of WPI film was also reported by Aziz et al. and Zhang et al. [71,72]. Furthermore, the addition of NCC increased the peak intensity at 2θ = 22° as a result of the trans-crystallization effect [43]. The trans-crystallization effect is proportional to the crystal orientation of the semi-crystalline matrix to the NCC [73]. The peak around 20–23° became broader, which indicated the good compatibility between WPI and glycerol [49]. The reflection at this peak shows the major structure was amorphous and displayed molecular miscibility and interaction between the component of biocomposite film [72]. The presence of this peak also indicated that the crystalline structure of the NCC material was preserved during the biocomposite film production. The compatibility between polymers and additives can result in the formation of homogeneous films with good mechanical and physical properties, which is an important factor for biocomposite films [74].

The crystallinity indexes of WPI and biocomposite film are shown in Table 11. The result shows that the presence of NCC in the WPI matrix offered a higher crystallinity index than the WPI film without NCC. These phenomena might be attributed to the formation of new hydrogen bonds NCC and WPI, which increased the degree of film crystallinity [75]. However, the crystallinity index in this study was higher than the study reported by Hafizulhaq et al. using cellulose fibers from bengkoang as filler in starch film and Carvalho et al. using nanocellulose from Eucalyptus sp. In WPI film [69,76]. A higher crystallinity index is probably due to the crystallization of glycerol as a plasticizer in WPI film. The storage time of film before analysis can also cause crystal growth in WPI film [77].

## 4. Conclusions

RSM-CCD was successfully used to optimize the physical and mechanical properties of a WPI composite film by optimizing the concentrations of glycerol and NCC. The results showed that both individual variables influenced the investigated responses for physical and mechanical properties of the biocomposite film. The model suggested that the optimum condition of the biocomposite film was at a 4% glycerol and 3.5% NCC concentration. The optimal model of independent variables was numerically predicted to achieve the desired thickness, tensile strength, and elongation at break values of 0.12 mm, 6.97 MPa, and 44.65%, respectively. The corresponding validation responses were thickness (0.13 mm), tensile strength (7.16 MPa), and elongation at break (39.10%). Additionally, the results in the XRD patterns show the increase in crystallinity index and the good compatibility between WPI, glycerol, and NCC. The formation of intramolecular hydrogen bonds between WPI, NCC, and glycerol also resulted in improved integration of NCC and glycerol into the protein matrix, as supported by FTIR and SEM analysis. The addition of NCC in optimized content also slightly improved the water resistance (WS and WVP) and decreased the transparency of the biocomposite film. Hence, the development of an optimized composite film formulation produced from WPI with the addition of glycerol and NCC can be an alternative as packaging materials to replace synthetic fibers in the future. However, further research is still needed in terms of investigating the biodegradability and storage ability, as well as the toxicity and antibacterial properties of composite films, so that the materials reinforced with natural fibers can produce better properties as packaging films.

## Figures and Tables

**Table 1:** Coded levels of variable glycerol and NCC.

Variable	Coded Levels				
	−α	−1	0 ^a^	+1	+α
X_1_: glycerol concentrations (%)	3.17	4	6	8	8.82
X_2_: NCC concentrations (%)	2.17	3	5	7	7.82

^a^ Center point; k = 2 (two independent variables); α = 1.414.

**Table 2:** The configuration of the response surface methodology–center composite design for biocomposite films.

Trial	Coded Variables		Actual Variables	
	X_1_	X_2_	X_1_ (%)	X_2_ (%)
1	0	0	6	5
2	−1	1	4	7
3	−1	−1	4	3
4	+α	0	8.82	5
5	−α	0	3.17	5
6	0	0	6	5
7	1	1	8	7
8	1	−1	8	3
9	0	−α	6	2.17
10	0	0	6	5
11	0	+α	6	7.82
12	0	0	6	5
13	0	0	6	5

**Table 3:** The responses of the central composite design parameters of the biocomposite films.

Trial	Independent Variables		Responses		
	Glycerol Concentrations (%)	NCCC oncentrations (%)	Thickness * (mm)	Tensile Strength * (MPa)	Elongation * (%)
1	6	5	0.23 ± 0.05	3.92 ± 0.98	35.88 ± 14.29
2	4	7	0.12 ± 0.01	3.60 ± 0.57	18.29 ± 3.84
3	4	3	0.09 ± 0.02	6.87 ± 0.22	40.29 ± 3.11
4	8.82	5	0.15 ± 0.03	1.64 ± 0.57	4.71 ± 1.70
5	3.17	5	0.09 ± 0.01	7.85 ± 0.11	41.13 ± 4.92
6	6	5	0.19 ± 0.03	4.03 ± 0.62	36.54 ± 11.83
7	8	7	0.24 ± 0.07	2.62 ± 0.57	6.71 ± 3.72
8	8	3	0.07 ± 0.01	1.96 ± 0.38	18.67 ± 7.32
9	6	2.17	0.12 ± 0.02	2.62 ± 0.57	42.92 ± 3.35
10	6	5	0.21 ± 0.03	4.12 ± 0.34	30.92 ± 9.54
11	6	7.82	0.16 ± 0.03	1.96 ± 0.23	14.80 ± 5.25
12	6	5	0.19 ± 0.03	4.92 ± 0.12	36.81 ± 13.93
13	6	5	0.21 ± 0.03	4.92 ± 0.12	36.87 ± 13.57

* Values are expressed as mean ± standard deviation.

**Table 4:** Variance analysis for the quadratic thickness model in biocomposite films.

Source	Sum of Squares	DF	Mean Squares	F Value	Prob > F
Model	0.0374	5	0.0075	13.40	0.0018 (significant)
Residual	0.0039	7	0.0006		
Lack-of-fit	0.0029	3	0.0010	3.86	0.1126 (not significant)
Pure error	0.0010	4	0.0003		
Total	0.0413	12			

Standard deviation = 0.0236; mean = 0.1583; R^2^ = 0.9054; and adequate precision = 8.6184.

**Table 5:** Variance analysis for the quadratic tensile strength model in biocomposite films.

Source	Sum of Squares	DF	Mean Squares	F Value	Prob > F
Model	40.05	5	8.01	22.26	0.0004 significant
Residual	2.52	7	0.3598		
Lack-of-fit	1.53	3	0.5099	2.06	0.2480 not significant
Pure error	0.9891	4	0.2473		
Total	42.57	12			

Standard deviation = 0.5999; mean = 3.93; R^2^ = 0.9408; and adequate precision = 15.1960.

**Table 6:** Variance analysis for the quadratic elongation at break model in biocomposite films.

Source	Sum of Squares	DF	Mean Squares	F Value	Prob > F
Model	2077.39	5	415.48	24.28	0.0003 significant
Residual	119.80	7	17.11		
Lack-of-fit	94.04	3	31.35	4.87	0.0802 not significant
Pure error	25.77	4	6.44		
Total	2197.19	12			

Standard deviation = 4.14; mean = 28.04; R^2^ = 0.9455; and adequate precision = 14.3242.

**Table 7:** The coefficients of regression and the probability values of approximate model for variables responses.

Term	Thickness		Tensile Strength		Elongation at Break	
	Coefficient	Probability	Coefficient	Probability	Coefficient	Probability
Constant	0.2064	0.0018	4.38	0.0004	35.41	0.0003
X_1_: Glycerol	0.0235	0.0262	−1.83	<0.0001	−10.59	0.0002
X_2_: NCC	0.0328	0.0057	−0.4423	0.0755	−9.21	0.0004
X_1_X_2_	0.0364	0.0178	0.9812	0.0136	2.51	0.2643
X_1_^2^	−0.0430	0.0020	0.2399	0.3265	−7.47	0.0021
X_2_^2^	−0.0352	0.0056	−0.9858	0.0034	−4.50	0.0241

**Table 8:** The acceptable range and validation for optimized biocomposite films.

Response	95% CI		95% PI		Validation
	Low	High	Low	High	Experimental Value
Thickness (mm)	0.06	0.15	0.04	0.18	0.13 ± 0.01
Tensile strength (MPa)	5.77	8.01	5.08	8.70	7.16 ± 0.33
Elongation (%)	38.01	53.48	33.28	58.22	39.10 ± 2.90

CI = confidence interval; PI = prediction interval.

**Table 9:** Transparency of of WPI and biocomposite films.

Film	Wavelength (nm)					
	300	400	500	600	700	800
WPI	0	6.53 ± 0.10	10.83 ± 0.06	15.53 ± 0.09	18.05 ± 0.55	22.51 ± 0.25
WPI/NCC/Glycerol	0	3.35 ± 0.09	4.99 ± 0.11	7.59 ± 0.03	11.24 ± 0.27	13.46 ± 0.46

**Table 10:** Water solubility and water vapor permeability of WPI and biocomposite films.

Film	WS (%) *	WVP (×10^−11^ g/m.s.Pa) *
WPI	30.21 ± 0.10	2.21 ± 0.10
WPI/NCC/Glycerol	27.15 ± 0.16	2.17 ± 0.08

* Value are expressed as mean ± standard deviation.

**Table 11:** Crystallinity index of WPI and biocomposite films.

Film	Crystallinity Index (%)
WPI	74.82
WPI/Glycerol	76.49
WPI/NCC/Glycerol	81.14

## Data Availability

The data presented in this study are available on request from the corresponding author.
